# The Influence of Thickness on Light Transmission for Pre- and Fully Crystallized Chairside CAD/CAM Lithium Disilicate Ceramics

**DOI:** 10.3390/ma17092045

**Published:** 2024-04-26

**Authors:** Franciele Floriani, Salahaldeen Abuhammoud, Silvia Rojas-Rueda, Amit Unnadkat, Nicholas G. Fischer, Chin-Chuan Fu, Carlos A. Jurado

**Affiliations:** 1 Department of Prosthodontics, The University of Iowa College of Dentistry and Dental Clinics, Iowa City, IA 52241, USA; 2 Division of Dental Biomaterials, The University of Alabama at Birmingham School of Dentistry, Birmingham, AL 35233, USA; 3 Division of Operative Dentistry, Department of General Dentistry, University of Tennessee Health Science Center, College of Dentistry, Memphis, TN 38103, USA; 4 Minnesota Dental Research for Biomaterials and Biomechanics, University of Minnesota School of Dentistry, Minneapolis, MN 55455, USA; 5 Department of Restorative Sciences, The University of Alabama at Birmingham School of Dentistry, Birmingham, AL 35233, USA

**Keywords:** ceramics, CAD-DAM, lithium disilicate, esthetic dentistry

## Abstract

Aim: This in vitro study aimed to compare the light-transmission properties of two chairside CAD/CAM lithium disilicate (LD) ceramics (a novel fully crystallized and a traditional pre-crystallized) across varying thicknesses. Materials and Methods: One hundred flat specimens were obtained from precrystallized (e.max CAD, Ivoclar Vivadent, Schaan, Liechtenstein) and fully crystallized (LiSi GC Block; GC, Tokyo, Japan) LD at five different thicknesses (0.5, 0.75, 1.0, 1.50 and 2.0 mm). All specimens were polished with a polishing system for lithium disilicate restorations following recommendations from the manufacturer. Light transmission was evaluated with a radiometer. The statistical analysis between e.max CAD and LiSi GC Block was performed using a Mann–Whitney test for each thickness at a significance level of 0.05 (*p* < 0.05), followed by a Kruskal–Wallis test to compare the light transmission between the thicknesses of e.max CAD and LiSi GC Block. Results: Light transmittance was significantly affected by ceramic thickness. The 0.5 mm thick specimens exhibited the highest transmittance values compared to all other groups, while a light transmittance of 0.00 was observed in the 2.0 mm thick specimens for both e.max CAD and LiSi GC Block. In the comparison between e.max CAD and LiSi GC Block according to thickness, there was a statistically significant difference exclusively between groups with a thickness of 1.50 mm (*p* = 0.002). Conclusions: Light transmission for pre- and fully crystallized CAD/CAM lithium disilicate ceramics only showed a statistical difference at the thickness of 1.50 mm (*p* = 0.002). E.max CAD demonstrated acceptable light transmission up to a thickness of 1.5 mm. Clinical Significance: A thickness of 2 mm for chairside CAD/CAM lithium disilicate ceramics, whether pre-crystallized or fully crystallized, necessitates the use of dual-cure resin luting cement due to reduced light transmission.

## 1. Introduction

Computer-aided design and computer-aided manufacturing (CAD/CAM) technology has revolutionized dentistry since its introduction in the 1980s, becoming increasingly prevalent in both clinical and laboratory settings [1]. Initially, this innovative technology was primarily utilized for fabricating inlay restorations chairside and delivering within the same day [2]. However, the scope of chairside CAD/CAM systems has expanded significantly over time [3,4,5]. CAD/CAM systems now enable clinicians to manufacture a wide array of materials for restorative procedures, including single and multi-unit crowns, onlays, inlays, veneers, fixed dental prostheses, implant prostheses, occlusal or bite guards, surgical guides, and diagnostic models. Furthermore, CAD/CAM technology has enhanced the efficiency of full mouth reconstructions and reduced the number of required appointments while increasing patient comfort [6].

Dental ceramics designed for chairside CAD/CAM systems have undergone significant advancements in recent years, showcasing remarkable longevity along with enhanced optical and mechanical properties [7]. Manufactured in a block format, milled dental ceramics are offered in sizes that are appropriate for a variety of restoration types, from single units to more extensive multi-unit applications. Ideally, these blocks should be capable of withstanding the rigors of the subtractive process and be easily finished prior to cementation [8]. Lithium disilicate has emerged as one of the most popular choices for chairside CAD/CAM ceramic blocks owing to its aesthetic appeal and high fracture resistance [9]. These materials are available in various translucency levels, including high, medium, and low. They are provided in a pre-sintered state, requiring the clinician to fully crystallize them in-office before cementation [10].

Lithium disilicate was introduced for CAD-CAM systems in the mid-2000s as pre-crystallized ceramic blocks, being marketed as IPS e.max CAD by Ivoclar Vivadent in Schaan, Liechtenstein. Many manufacturers have since developed and marketed materials based on IPS e.max. In some cases, they have added novel characteristics, such as the option to vary translucency within a single block through different sintering parameters, such as temperature and time (Amber Mill; Hass Bio, Gangwon-do, Republic of Korea) [11]. These materials are milled in a pre-crystallized state and become fully crystallized after sintering [12]. A fully crystallized lithium disilicate (LiSi GC Block; GC, Tokyo, Japan) material was somewhat recently brought to market. This material is unique, as the typical sintering process in-office after milling is not necessary, although additional sintering treatments are possible.

Supplemental thermal treatment is often an essential step in the fabrication of dental restorations using lithium disilicate (LD) through the CAD/CAM process whenever specific stains are needed in order to mimic adjacent natural dentition [13]. The manufacturing of CAD/CAM LD glass−ceramics encompasses a series of requirements, including industrial casting of blocks for distribution, milling and crystallization [14]. During milling, the ceramic is in a partially crystallized lithium metasilicate (LM) state. This state enhances cutting efficiency and simplifies the process due to the moderate strength and hardness of LM [15]. Following the milling, crystallization through thermal steps induces significant structural transformations in the LM ceramic, consequently promoting growth and evolvement of the LD crystals [16,17]. This supplemental thermal treatment affects the optical and mechanical properties of LD, thereby increasing the long-term success of ceramic treatments in dental clinics [18].

Translucency, a key element in the aesthetic outcome of dental ceramics, is often evaluated through direct light transmittance, a reliable method for assessing the translucency and opacity of aesthetic restorative materials [19]. At the same time, mechanical strength plays a vital role in the clinical success and longevity of dental restorations [20]. The thermal treatment, which involves temperature-driven dissolution of LD, results in variations in crystal size, thereby influencing the optical and mechanical properties of the ceramics [21]. The optical properties of ceramic restorations are an important key factor, as they significantly influence the polymerization reaction of resin luting cement due to light interactions during the bonding process [22]. Recent studies have shown encouraging results regarding the mechanical properties of this novel pre-crystallized ceramic [23]; however, there is a lack of research evaluating the degree of light transmission in these newly developed chairside CAD/CAM lithium disilicates. Understanding the extent of light transmission through a dental ceramic is vital to enabling clinicians to make an informed choice between light-cured and self-cured resin cements based on the specific characteristics of the ceramic material in use [24].

The thickness of ceramic materials determines the extent of tooth reduction and significantly influences the optical and mechanical properties. Previous studies have shown that the translucency of dental ceramics, including glass ceramics and zirconia, is markedly affected by their thickness, with translucency decreasing as thickness increases [18,25]. Furthermore, an increase in thickness has been observed to enhance the flexural strength in a variety of ceramic materials, such as glass-infiltrated aluminum oxide, lithium-disilicate-reinforced glass−ceramics, as well as yttrium-stabilized zirconia, irrespective of aging processes [26]. Conversely, another study noted a minimal impact on overall flexural strength due to increased thickness in two specific dental ceramics: Dicor^®^ (Corning Glass Works, Corning Inc., Corning, NY, USA) (a fine-grained glass−ceramic) and Cerestore^®^ (a magnesia-alumina spinel core ceramic) (Cerestore, Johnson & Johnson, New Brunswick, NJ, USA) [21]. Notably, a significant proportion of failures in these ceramics were initiated in areas where the thickness exceeded 1.5 mm [27]. Information about the variation in optical and mechanical properties at different thickness levels is crucial for dental clinicians in regard to selecting the most suitable clinical cases for using LD and determining the appropriate degree of tooth reduction. However, few studies in the literature have evaluated the optical properties of different types of LD glass−ceramics for CAD/CAM, particularly for precrystallized (sintering process is mandatory) and fully crystallized (sintering process is not mandatory) forms with diverse thicknesses.

Therefore, the purpose of this study is to evaluate the light transmission of pre- and fully crystallized chairside CAD/CAM lithium disilicate glass–ceramics of various thicknesses. To achieve this purpose, pre-crystallized (e.max CAD, Ivoclar Vivadent, Schaan, Liechtenstein) and fully crystallized LD (LiSi GC Block; GC, Tokyo, Japan) specimens were evaluated at five different thicknesses (0.5, 0.75, 1.0, 1.50 and 2.0 mm). The null hypotheses were that (1) there is no difference in light transmission between e.max CAD and LiSi GC Block within the same thickness of samples (0.50 mm, 0.75 mm, 1.00 mm, 1.50 mm, 2.00 mm), and (2) there is difference in light transmission within e.max CAD and LiSi GC Block at different thicknesses.

## 2. Materials and Methods

The sample size calculation was based on a previous study by Jurado et al., 2021 [28]. Blocks of novel chairside CAD/CAM lithium disilicate blocks LiSi GC Block (GC, Tokyo, Japan) and e.max CAD (Ivoclar Vivadent, Schaan, Liechtenstein) were acquired, and one hundred flat specimens were obtained with a low-speed precision cutting machine (Isomet; Buehler, Lake Bluff, IL, USA) under constant water cooling and divided into ten groups (*n* = 10/group). All prepared e.max CAD specimens were sintered following the manufacturers’ temperature and time recommendations for high translucency. The pre-crystallized e.max CAD (Ivoclar Vivadent, Schaan, Liechtenstein) samples were fired in a dental furnace; 5 min at 840 °C for e.max CAD at 840 °C, while LiSi GC Block (GC, Tokyo, Japan) is a fully sintered material that does not require in-office sintering process.

Subsequently, all one hundred flat specimens with different thicknesses were polished using a commercially available polishing system for lithium disilicate restorations (K0293 IPS e.max Chairside Adjustment and Polishing System, Brasseler USA, Savannah, GA, USA) based on recommendations from the manufacturer. Every sample was tested for light transmission with a LED light-curing unit (1000 mW/cm^2^; VALO Cordless, Ultradent, South Jordan, UT, USA) using a curing radiometer (LM-1 LED Light Meter, Guilin Woodpecker Medical Instrument, Guilin, China). This enabled the average light intensity through the specimen in mW/cm^2^ to be recorded.

A scanning electron microscope (SEM, TM3000, Hitachi High Technology, Tokyo, Japan) was utilized for microstructural analysis. Prior to observation, a thin gold coating was applied in a sputter coater (Quick Coater Type SC-701, Sanyu Electro, Tokyo, Japan) to enhance the electron conductivity of the specimens. This process allowed for the detailed examination of ceramic-surface microstructures at different magnification levels.

### Statistical Analysis

To evaluate the differences in light-transmission properties between e.max CAD and LiSi GC Block across various thicknesses, the descriptive data included 100 samples, and the mean, standard deviation (SD), median, 25th and 75th percentiles, as well as the values of minimum and maximum were compiled. The measurement variables did not meet the normality assumption based on Kolmogorov–Smirnov testing. The comparison between E.max CAD and LiSi GC Block was performed using a Mann–Whitney test for each thickness at a significant level of 0.05 (*p* < 0.05). This was aimed at identifying any significant disparities between the two materials at each specific thickness. Subsequently, a Kruskal–Wallis test facilitated a comprehensive comparison on light transmission across all thicknesses for both materials. To ensure the accuracy of our findings amidst multiple comparisons, adjustments were made using the Bonferroni correction.

## 3. Results

Light transmission of 100 samples, with the mean, standard deviation (SD), median, and the percentiles of 25th and 75th, as well as the minimum and maximum values for all ten groups (*n* = 10/group), are represented in Table 1.

The comparison between E.max CAD and LiSi GC Block was performed using a Mann–Whitney test for each thickness. The transmittance was significantly affected by the ceramic thickness. The 0.5 mm thick specimens showed the highest transmittance values compared to other groups, and 0.0 light transmittance was observed with the 2.0 mm thick (Table 1) specimens for both e.max CAD and LiSi GC Block. In a comparison between e.max CAD and LiSi GC Block in regard to thickness, there was a statistically significant difference between glass−ceramics e.max CAD and LiSi GC Block only for the thickness of 1.50 mm (*p* = 0.002) (Table 2).

There were statistically significant differences (Kruskall−Wallis test= 41.58; *p* < 0.001) in e.max CAD groups with different thicknesses. The 2.00 mm thickness showed lower light transmission than 1.00 mm (*p* = 0.007), 0.75 mm (*p* < 0.001) and 0.50 mm (*p* < 0.001). The 1.50 mm thickness showed lower light transmission than 0.50 mm (*p* < 0.001) (Table 3).

There were statistically significant differences between different thicknesses of LiSi GC Block (Kruskall−Wallis test = 45.81; *p* < 0.001) (Table 4).

The 2.00 mm thickness showed a lower light transmission than 0.75 mm (*p* < 0.001) and 0.50 mm (*p* < 0.001). The 1.50 mm thickness showed a lower light transmission than 0.75 mm (*p* = 0.003) and 0.50 mm (*p* < 0.001). The 1.00 mm thickness showed a lower light transmission than 0.50 mmm (*p* = 0.023).

**Figure 1:**
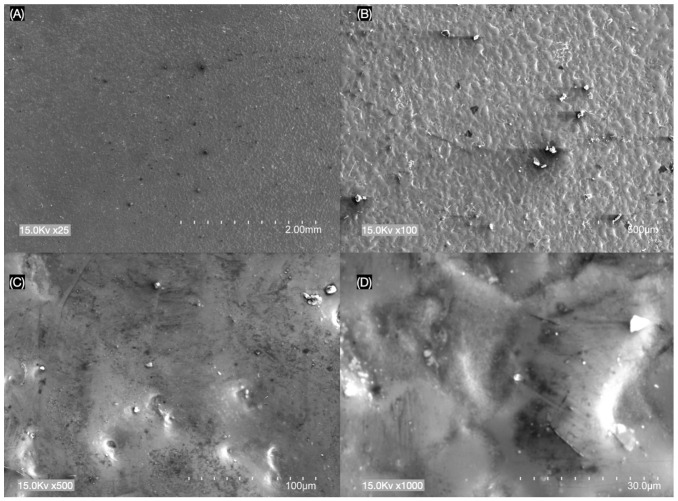
Pre-crystallized lithium disilicate (e.max CAD) surface with (**A**) ×25, (**B**) ×100, (**C**) ×500 and (**D**) ×1000 magnification.

**Figure 2:**
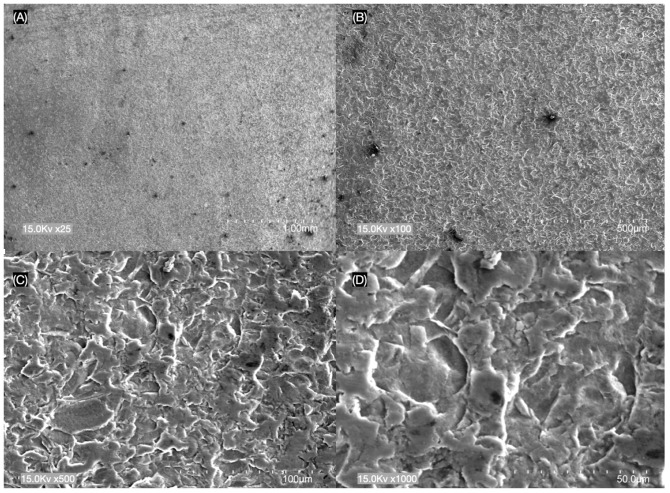
Fully crystallized lithium disilicate (LiSi GC Block) surface with (**A**) ×25, (**B**) ×100, (**C**) ×500 and (**D**) ×1000 magnification.

The results of SEM analysis showed different surface morphologies between e.max CAD and LiSi GC Block at (A) ×25, (B) ×100, (C) ×500 and (D) ×1000 magnifications (Figure 1 and Figure 2). The LiSi GC Block ceramic surface specimens showed more porosities and rougher appearances across their surfaces compared to the e.max CAD group.

## 4. Discussion

This study evaluated light transmission for a novel chairside CAD/CAM fully crystallized lithium disilicate ceramic LiSi GC Block (GC, Tokyo, Japan) compared to a pre-crystallized lithium disilicate ceramic e.max CAD (Ivoclar Vivadent, Schaan, Liechtestein) at five different thicknesses (0.5, 0.75, 1.0, 1.50 and 2.0 mm). The results showed that light transmission changes significantly as the thickness changes; therefore, the null hypothesis (1) that there is no difference in light transmission between e.max CAD and LiSi GC Block within the same thickness of samples (0.50 mm, 0.75 mm, 1.00 mm, 1.50 mm, 2.00 mm) was rejected. The secondary hypothesis, (2) that there is difference in light transmission within e.max CAD and LiSi GC Block with different thicknesses, was accepted.

In this study, the specimens were prepared with thicknesses to simulate clinical scenarios between 0.5, 0.75, 1.0, 1.50 and 2.0 mm. In an anterior restoration, for example, the ceramic thickness might be approximately 1 mm at the margins, increasing to between 1.5 to 2.0 mm at the incisal edge or cusp areas [28]. The radiometer utilized was capable of accurately measuring light intensity transmitted through the prepared blocks from the LED light-curing unit, considering both the radiating surface area and emitted light.

The transmission results of this study align with findings from numerous previous studies, which all consistently demonstrated a decrease in translucency as the material thickness increased [29,30]. This phenomenon aligns with the Beer–Lambert law, which suggests that materials with greater thickness absorb more light, resulting in lower light transmission [31]. When a significant amount of light is passed through a ceramic material and is diffusely reflected, it gives the material an opaque appearance. Additionally, materials composed of fewer particles per unit volume generally exhibit reduced light scattering and decreased opacity [32]. All this evidence suggests that the thickness of restorations from lithium disilicate blocks significantly affects the polymerization reactions of resin luting cements during bonding due to light transmission decreasing with increasing thicknesses above 2.0 mm [31]; however, more evidence is needed to support this conclusion with CAD/CAM materials. Previous research has indicated that the hardness of dual-cure resin luting cement shows no significant difference when light-cured at 1.0 mm or 2.0 mm with thicknesses of conventional lithium disilicate [33]. Moreover, manufacturers recommend fabricating chairside CAD/CAM ceramic restorations with a thickness of no more than 2 mm [30]. As a result, light transmission through these restorations may not significantly impede the photopolymerization reactions of dual-cure resin luting cement under 2.0 mm, despite a reduction in light transmission.

A previous study studied the effect of different opacities and thicknesses of lithium disilicate on the degree of conversion (DC) of two different resin cements, using lithium disilicate samples with either high translucency (HT), medium opacity (MO) or low translucency (LT) while using IPS e. max CAD in five differing thicknesses. Increasing thickness caused a decrease in the DC in both cements under all conditions tested. Additionally, comparing the opacities, the more translucent ceramics exhibited larger DC values than less translucent ceramic samples [34]. Importantly, dual-cure resin cements have more variation in color than light-polymerized materials [35]. An additional consideration, as it relates to color variation, is the stump or build-up shade.

However, factors beyond restoration thickness must be considered. Factors like the light source, shade of the lithium disilicate blocks, chemical composition and curing mode of the resin luting cements, distance to the restoration surface, as well as the light intensity of the curing unit also affect the polymerization reactions of all dual-cure resin luting cements. Consequently, it is crucial to recognize that restoration thickness might interact with these variables to significantly impact polymerization. These findings suggest that clinicians should keep in mind the influence of CAD/CAM lithium disilicate restorations on light transmittance and the thickness of preparation [28].

Limited data are available evaluating the novel chairside fully crystallized LiSi GC ceramic. A previous study investigated the fracture toughness and structure of the glass−ceramics LiSi GC and e.max CAD at varying degrees of translucency. Translucency levels of both lithium disilicate ceramics did not alter the material fracture toughness. The e.max CAD fracture toughness was significantly higher than LiSi [36]. Another study compared the mechanical and optical characteristics of lithium disilicate (Initial LiSi Block, GC), lithium disilicate/lithium aluminum silicate (Tessera, Dentsply/Sirona), 4Y polycrystalline-stabilized zirconia (IPS e.max ZirCAD MT, Ivoclar Vivadent; Katana STML, Kuraray; YZ ST, VITA) and IPS e.max CAD (Ivoclar Vivadent). Generally, the lithium-disilicate-based ceramic materials showed better optical properties and worse mechanical properties than the zirconia-based ceramic materials [37]. However, no one to date has evaluated the light transmission with different thicknesses to understand the clinical acceptable measurements of the preparation, as well as the importance of dual-cure cement instead of light-curing cement.

In the present study, SEM observations showed that LiSi GC ceramic specimens exhibited more surface texture and irregularities compared to e.max CAD (Figure 1 and Figure 2). LiSi showed dense crystals of approx. 1 to 1.5 μm in size, while e.max CAD showed long rod-like crystals of approx. 3 to 4 μm in size and LiSi GC showed more porosities and rougher appearances across their surfaces compared to the e.max CAD group. The observed differences likely influenced the light intensity transmitted, as the e.max CAD reduced roughness and increased the transmittance of light through the materials.

Numerous companies are currently developing novel CAD/CAM lithium disilicate blocks for chairside applications, claiming enhanced and modified optical properties compared to conventional equivalents. For instance, the LiSi GC, a novel lithium disilicate block, demonstrated clinically acceptable light transmittance in four of the five different thicknesses (0.5, 0.75, 1.0, 1.50, but not 2.0 mm) specimens, indicating the importance of using a dual-cure resin luting cement for 2.00 mm. However, other new materials may exhibit different properties. This underscores the importance for clinicians to rationalize their decision making and clinical approaches to the specific properties of each material. Despite these advancements, there is still missing and incomplete data on the properties of these novel materials. Consequently, further studies are required to comprehensively evaluate the range of new chairside CAD/CAM lithium disilicate ceramics offered on the market today.

This study aimed to guide clinicians in understanding the degree of light transmission in two of the most common chairside CAD/CAM lithium disilicate ceramics on the market. However, limitations were identified that are related to the use of only two different brands of lithium disilicate, while more brands have recently become available on the market. It would also be valuable to compare lithium disilicate with novel translucent zirconia ceramics using the same methodology; additionally, it would be valuable to compare the light transmission of the ceramic cemented with different resin cements. Lastly, evaluating more devices for light transmission may be interesting as well, as the device used in this study only provides values with multiples of 25.

## 5. Conclusions

Within the limitations of this in vitro study, it was possible to conclude that:

1. The light transmission of the novel chairside CAD/CAM lithium disilicate ceramic LiSi GC Block decreases as the thickness increases.
2. The light transmission of the novel chairside CAD/CAM lithium disilicate ceramic e.max CAD decreases as the thickness increases.
3. Light transmission for pre- and fully crystallized chairside CAD/CAM lithium disilicate ceramics showed a statistically significant difference at the thickness of 1.50 mm (*p* = 0.002).
4. E.max CAD showed acceptable light transmission at up to 1.5 mm of thickness.
5. Both pre- and fully crystallized chairside CAD/CAM lithium disilicate ceramics with 2 mm of thickness should be cemented with dual-cure resin luting cements due to the reduction in light transmission.

## Figures and Tables

**Table 1:** Summary of the light transmission data.

Thickness	N	Mean	SD	Median	P25	P75	Minimum	Maximum
e.max CAD								
0.50 mm	10	357.5	40.9	350.0	343.8	362.5	300.0	450.0
0.75 mm	10	272.5	69.2	275.0	200.0	331.3	150.0	350.0
1.00 mm	10	225.0	57.7	212.5	187.5	300.0	150.0	300.0
1.50 mm	10	160.0	17.5	150.0	150.0	175.0	150.0	200.0
2.00 mm	10	0.0	0.0	0.0	0.0	0.0	0.0	0.0
LiSi GC Block								
0.50 mm	10	372.5	32.2	375.0	350.0	400.0	300.0	400.0
0.75 mm	10	300.0	33.3	300.0	287.5	312.5	250.0	350.0
1.00 mm	10	197.5	29.9	200.0	187.5	206.3	150.0	250.0
1.50 mm	10	57.5	74.6	0.0	0.0	150.0	0.0	150.0
2.00 mm	10	0.0	0.0	0.0	0.0	0.0	0.0	0.0

Note. SD: standard deviation. P: percentile.

**Table 2:** Comparison between e.max CAD and LiSi GC Block according to the thickness.

e.Max CAD vs. LiSi GC Block	Mann–Whitney Test	*p* Value
0.50 mm	68.50	0.165
0.75 mm	60.00	0.481
1.00 mm	36.00	0.315
1.50 mm	10.50	0.002
2.00 mm	50.00	1.000

**Table 3:** Multiple comparison between the thicknesses of e.max CAD.

e.Max CAD	Test Statistic	Standard Error	*p* Value ^a^
2.00 mm–1.50 mm	12.200	6.445	0.584
2.00 mm–1.00 mm	21.800	6.445	0.007
2.00 mm–0.75 mm	27.450	6.445	<0.001
2.00 mm–0.50 mm	38.550	6.445	<0.001
1.50 mm–1.00 mm	9.600	6.445	1.000
1.50 mm–0.75 mm	15.250	6.445	0.180
1.50 mm–0.50 mm	26.350	6.445	<0.001
1.00 mm–0.75 mm	5.650	6.445	1.000
1.00 mm–0.50 mm	16.750	6.445	0.094
0.75 mm–0.50 mm	11.100	6.445	0.850

Note: Asymptotic significances (2-sided tests) are displayed. The significance level is 0.05. ^a.^ Significance values have been adjusted by the Bonferroni correction for multiple tests.

**Table 4:** Multiple comparisons between the thicknesses of LiSi GC Block.

LiSi GC Block	Test Statistic	Standard Error	*p* Value ^a^
2.00 mm–1.50 mm	4.300	6.390	1.000
2.00 mm–1.00 mm	16.800	6.390	0.086
2.00 mm–0.75 mm	27.600	6.390	<0.001
2.00 mm–0.50 mm	36.300	6.390	<0.001
1.50 mm–1.00 mm	12.500	6.390	0.504
1.50 mm–0.75 mm	23.300	6.390	0.003
1.50 mm–0.50 mm	32.000	6.390	<0.001
1.00 mm–0.75 mm	10.800	6.390	0.910
1.00 mm–0.50 mm	19.500	6.390	0.023
0.75 mm–0.50 mm	8.700	6.390	1.000

Note: Asymptotic significances (2-sided tests) are displayed. The significance level is 0.05. ^a.^ Significance values have been adjusted by the Bonferroni correction for multiple tests.

## Data Availability

Data are contained within the article.
